# Autoimmune gastritis

**DOI:** 10.1007/s10354-016-0515-5

**Published:** 2016-09-26

**Authors:** Stefanie Kulnigg-Dabsch

**Affiliations:** Department of Medicine III, Medical University of Vienna, Waehringer Guertel 18–20, Vienna, 1090 Austria

**Keywords:** Iron deficiency, Vitamin B 12 deficiency, Autoimmune gastritis, Chronic atrophic gastritis, Gastric cancer, Eisenmangel, Vitamin-B12-Mangel, Autoimmungastritis, Chronisch atrophische Gastritis, Magenkarzinom

## Abstract

Autoimmune gastritis is a chronic inflammatory disease with destruction of parietal cells of the corpus and fundus of the stomach. The known consequence is vitamin B12 deficiency and, consequently, pernicious anemia. However, loss of parietal cells reduces secretion of gastric acid which is also required for absorption of inorganic iron; thus, iron deficiency is commonly found in patients with autoimmune gastritis. This usually precedes vitamin B12 deficiency and is found mainly in young women. Patients with chronic iron deficiency, especially those refractory to oral iron therapy, should therefore be evaluated for the presence of autoimmune gastritis.

## Zusammenfassung

Die Autoimmungastritis ist eine chronisch-entzündliche Erkrankung mit Verlust der Parietalzellen im Corpus und Fundus des Magens. Bekannte Folgen sind ein Vitamin-B_12_-Mangel und folglich eine perniziöse Anämie. Der Verlust der Parietalzellen führt jedoch auch zu einer Reduktion der Magensäure, die für die Aufnahme von anorganischem Eisen benötigt wird. Daher ist Eisenmangel eine häufige Diagnose bei Patienten mit Autoimmungastritis. Oft findet man diesen vor dem Auftreten eines Vitamin-B_12_-Mangels, insbesondere bei jüngeren Frauen. Patienten mit chronischem Eisenmangel sollten daher auf Marker einer Autoimmungastritis untersucht werden, vor allem wenn sie nicht auf eine orale Eisensubstitution ansprechen.

## Introduction

Autoimmune gastritis (AIG) is a chronic inflammatory disease of the stomach, finally presenting with atrophy of the mucosa. It is very important to note that chronic atrophic gastritis is not synonymous with AIG, as atrophy of the mucosa is the final result of a chronic inflammatory disease independent of etiology, which is mainly Helicobacter pylori gastritis or AIG. In contrast to Helicobacter pylori-, stress-, or drug-induced gastritis, inflammation and continuous atrophy is restricted to corpus and fundus in AIG. This is due to the fact that the autoimmune reaction in AIG targets parietal cells. Parietal cells are epithelial cells located in the glands of the corpus and fundus but not in the antrum, and produce hydrochloric acid and intrinsic factor. The acidification of the stomach is primarily managed by the gastric H+/K+ ATPase, the proton pump, which is the causative autoantigen and which is recognized by CD4+ T cells [1]. The chronic inflammation leads to an atrophy of the mucosa, with a decrease and final total loss of parietal cells during progression of disease. This results in increased pH of the stomach and loss of intrinsic factor, which is produced by parietal cells. Intrinsic factor is required for uptake of vitamin B12 and vitamin B12 deficiency (pernicious anemia) is a known consequence of AIG. As early as 1909, “achylia gastrica” was identified as the underlying cause of iron deficiency by Faber [2]. Thereafter it was long ignored, until the end of the last millennium when several authors addressed the question of iron deficiency and AIG in their studies [3, 4].

## AIG and iron deficiency

The total iron content of a healthy adult is about 4 g. Two thirds are bound in hemoglobin; the vast majority of that remaining is stored in ferritin in all types of cells. Only a few milligrams are actively circulated via binding to transferrin. Physiological daily iron loss via shredded cells of skin and gastrointestinal mucosa as well as hair loss is about 1–2 mg; the same amount is taken up by enterocytes of the duodenum and upper jejunum. Iron metabolism is only regulated via uptake as there is no active excretion mechanism. A distinction is drawn between uptake of heme (meat) and non-heme iron. Heme iron is taken up via a heme receptor [5] into the enterocyte, where iron is then released. Non-heme iron uptake is more complex and about 80 % of dietary iron is non-heme iron [6]. Inorganic iron in all kinds of food is in its ferric (Fe^3+^) form and must be reduced to ferrous (Fe^2+^) before it can enter the enterocytes via the divalent metal transporter 1. This is managed by enzymatic reduction (duodenal cytochrome B), but several other factors also reduce iron, of which the most important is gastric acid.

Iron deficiency occurs if there is an imbalance of iron uptake and iron loss. The most common cause is increased loss due to acute or chronic bleeding (gastrointestinal, GI, bleeding; augmented menstrual bleeding). Besides loss of iron, decreased iron absorption is causative of iron deficiency. This might be due to inflammation at the site of iron uptake, which is found in Crohn’s disease and also coeliac disease. The importance of normal gastric secretion and acidity for uptake of iron has been reported in several studies [7–9]; also patients with gastrectomy are known to suffer from iron deficiency anemia [10]. Abnormal gastric secretin in patients with AIG is well described [2, 11] and led to the term “achylia gastrica”. Besides the absence of gastric acid, ascorbic acid in food sources is also important for iron uptake. In AIG this was found to be reduced. It is hypothesized that ascorbic acid is destroyed due to the higher gastric pH [12]. On the other hand, nutritional iron (ferric and ferrous) is generally bound to proteins and needs to be released for uptake. Gastric acid is therefore needed. Iron deficiency as a result of AIG was already described more than a century ago [13, 14]. Cook et al. evaluated the absorption of dietary non-heme iron in controls vs. AIG patients and found a reduced iron uptake (19.8 vs. 35 %) [8]. In a subgroup of patients the effect of addition of gastric juice to the test meal in iron absorption was examined: an increased iron uptake of more than twofold could be found, whereas neutralized gastric juice did not change iron absorption. Likewise, AIG patients are typically refractory to oral iron therapy [4, 15].

In summary, decreased uptake of inorganic iron due to missing reduction of ferric iron, missing degradation of iron–protein complexes as a result of lack of gastric acid, and reduced levels of ascorbic acid are the likely causes of iron deficiency in AIG.

Vitamin B12 deficiency in AIG is not only caused by loss of intrinsic factor if parietal cells are destroyed, but is also due to loss of gastric acid, which is needed for release of the vitamin from food sources. Pernicious anemia is a rather late finding in AIG and diagnosed mainly in elderly patients [11]. This might be explained by minimal turnover (2–3 µg per day) with large stores (2–5 mg) on one hand, and on the other hand, by the fact that vitamin B12 absorption is additionally reduced with age.

Although AIG impairs both iron and vitamin B12 uptake, iron deficiency will be found at a younger age and many years before the development of pernicious anemia [11], as in younger (premenopausal) woman menstrual blood loss (and pregnancies) is an additional burden to iron metabolism. This is supported by results of several epidemiologic studies [3, 4, 15, 16].

## Clinical presentation

Symptoms vary during the course of disease (Table 1). Contrary to other types of gastritis (B or C), gastritis-associated symptoms such as pain are not in the foreground; furthermore, AIG patients have no risk of developing a gastric or duodenal ulcer. Achlorhydria itself leads to symptoms such as delayed gastric emptying, small intestinal (and gastric) bacterial overgrowth, and an increase in GI infections such as clostridium difficile colitis. The main clinical manifestation of AIG is known to be pernicious anemia; however, Hershko et al. showed in 2006 that the predominant hematologic manifestation of patients with AIG is iron deficiency anemia, as defined by microcytic anemia which was present in 50 % of patients [16]. Symptoms of iron deficiency arise independently of and prior to anemia-related symptoms, and include fatigue, restless legs syndrome, brittle nails, hair loss, impaired immune function, and impaired wound healing. Anemia (irrespective of etiology) results in shortness of breath, dizziness, tachycardia, lightheadedness, and decreased cognitive and physical function. In pregnancy it can lead to poor pregnancy outcome with early birth and underweight newborns. Very typical for AIG is the refractoriness to oral iron therapy.

Besides hematologic symptoms, patients who developed vitamin B12 deficiency will suffer from gastrointestinal and neurological complaints such as malabsorption, diarrhea, weight loss, glossitis, peripheral numbness, paresthesia with subsequent development of weakness, and ataxia. Furthermore, mental disturbances ranging from forgetfulness to psychosis may arise. Untreated pernicious anemia is fatal [11].

As AIG is associated with other autoimmune diseases, symptoms such as fatigue may also be due to thyroid hypofunction in the presence of autoimmune thyroiditis (Hashimoto’s disease). Patients newly diagnosed with AIG should therefore be screened for thyroid diseases. More rarely, diabetes type 1 or Addison’s disease is found, and should be taken into account if symptoms do not resolve after treatment of deficiencies.

## Epidemiology

Data on incidence are difficult to obtain, as most patients will be asymptomatic for years due to late and unspecific development of symptoms. The study of Zhang found a prevalence of 19.5 % of positive parietal-cell antibodies (PCA) in patients (recruited during general health check-up at general practitioner), with numbers increasing with age and in patients positive for Helicobacter pylori, but with no difference between the genders [17]. Atrophic gastritis as defined by serological testing (pepsinogen I and pepsinogen I/II ratio) was found in 46 % of patients positive for PCA vs. 18 % in those negative. However, only patients between the age of 50 and 74 years were included (mean 62 years); therefore, this study might overestimate the number as prevalence seems to increase with age. The study of Cabrera included randomly selected individuals from the Canary Islands between the age of 18 and 75 years. A total of 7.8 % were tested positive for PCA; higher numbers were found for women, with no age difference [18]. There are a number of other studies assessing the prevalence of pernicious anemia or chronic atrophic gastritis; however, these do not reflect the true prevalence for AIG, as pernicious anemia is the end stage of disease and chronic atrophic gastritis is not exclusively due to AIG but mainly due to Helicobacter pylori infection.

Helicobacter pylori has even been discussed as trigger for AIG because of the molecular mimicry between *H. pylori *antigens and the gastric H/K ATPase [19, 20]. Although several studies have examined this issue, the role of Helicobacter pylori in pathogenesis remains unclear.

In contrast, the association between AIG and other autoimmune diseases is clearly known. Especially in patients with autoimmune thyroiditis, where the incidence increases to up to ~35 % [21, 22]. But also other autoimmune diseases such as diabetes type I, Addison’s disease, and vitiligo show an increased risk [23–25].

Epidemiologic data on the prevalence of AIG in patients with (iron deficiency) ID are available from five studies (Table 2): Dickey et al. studied 41 patients with iron deficiency anemia (IDA). Atrophic gastritis diagnosed by histological evaluation was found in 20 % (*n* = 8) of patients, of whom 6 had detectable antibody levels against intrinsic factor or parietal cells, one patient had detectable Helicobacter pylori, and the last patient had neither, resulting in a prevalence of AIG of 15 % [3]. In the study of Hershko, a higher incidence of AIG in patients with IDA could be found. One hundred and fifty patients with iron deficiency anemia of unknown etiology were included. Positive levels of PCA together with hypergastrinemia pointing to AIG were diagnosed in 27 % [4]. The study of Annibale evaluated the presence of atrophic gastritis in IDA (26 %); however, unfortunately, no characterization of atrophy (antrum or body/fundus) or further testing for AIG was done [15]. As 13 of the 19 patients (total 18 %) were positive for Helicobacter pylori, the incidence for AIG might be lower. In a British study, 12 of 44 (27 %) anemic patients with iron deficiency were positive for PCA [26]. The latest study of Kulnigg-Dabsch evaluated the cause of iron deficiency (with or without anemia) in 409 patients and found positive PCA in 18.5 %. Patients with positive PCA had lower hemoglobin and ferritin levels and were more prone to restless legs syndrome [27]. Furthermore, patients with IDA and AIG seems to be younger and predominantly female [16].

**Table 1:** Prevalence of autoimmune gastritis in patients with iron deficiency

Study	Number	Prevalence of autoimmune gastritis in iron deficiency		
		Patients	Test	Prevalence (%)
Dickey et al. [3]	41	Iron deficiency anemia	Histology and pos PCA/IFA	15
Hershko et al. [4]	150	Iron deficiency anemia	↑ Gastrin and pos PCA	27
Annibale et al. [5]	71	Iron deficiency anemia	Histology (body atrophy)	27
Kaye et al. [26]	156 44	Iron deficiency anemia	Histology (body atrophy) Pos PCA	26 27
Kulnigg-Dabsch et al. [27]	409	Iron deficiency	Pos PCA	19

*PCA parietal cell antibodies, IFA* intrinsic factor antibodies, *pos* positive

**Table 2:** Symptoms of iron deficiency, vitamin B12 deficiency, and anemia

Symptoms		
Iron deficiency	Vitamin B12 deficiency	Anemia
Fatigue	*Neurological*	Shortness of breath
Restless legs syndrome	Peripheral neuropathy	Dizziness
Attentiveness disorder	Myelopathy	Tachycardia
Brittle nails	Spinal ataxia	Decreased physical function
Hair loss	Weakness	Decreased cognitive function
Sleeping disorder	Depression	–
–	*Gastrointestinal*	–
–	Glossitis	–
–	Malabsoprtion	–
–	Diarrhea	–

*ID* iron deficiency, *IDA* iron deficiency anemia

## Diagnosis of AIG

**Fig. 1:**
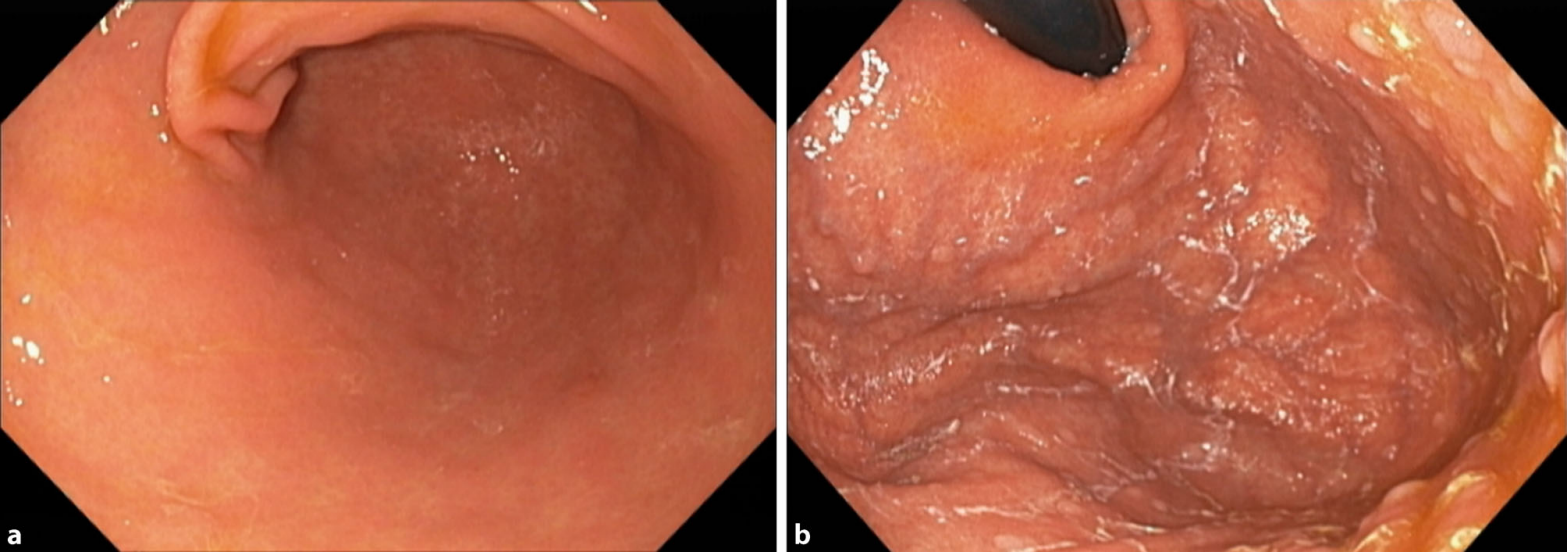
Endoscopic picture of autoimmune gastritis. **a** Incipient atrophy of corpus. **b** Mucous covering of gastric mucosa, pseudopolyps in corpus and fundus

**Fig. 2:**
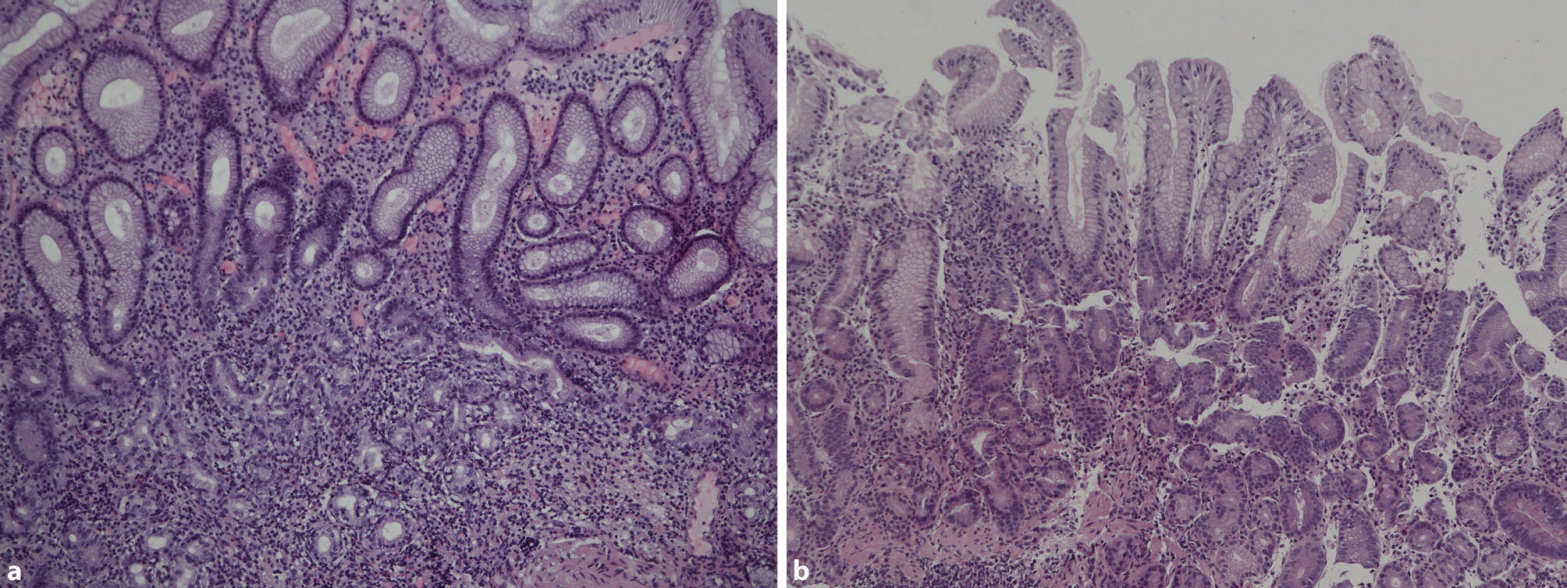
Histologic picture of end stage autoimmune gastritis (hematoxylin and eosin staining, X200). **a** Total loss of parietal cells with moderate inflammatory cells consisting of mainly mononuclear cells but also eosinophil granulocytes. **b** Foveola and gland bodies with total loss of parietal cells and only discrete inflammatory reaction

Gold standard for diagnosis of AIG is gastroscopy, with separately collected biopsies of antrum and corpus with typical histological findings [28]. The endoscopic appearance of AIG may not be different from the healthy situation during early phases of disease; however, with increasing loss of the oxyntic mucosa, pseudopolyps might be seen which mimic relatively normal mucosa while the surrounding is atrophic [29]. If extensive atrophy is present, rugal folds are flattened, submucosal vessels may be visible, and pseudopolyps or polyps (hyperplastic or adenomatous) might be present ([30, 31]; Fig. 1). Typical histological findings change during the course of disease. In early phases, lymphocytic and plasma cell infiltration of the oxyntic mucosa is found, mainly multifocal with accentuation in the deeper, glandular portion [32]. Oxyntic glands might be destroyed fragmentary, and parietal cells show pseudohypertrophic changes. As these features are non-specific, pathologists might misinterpret findings without knowledge of serum markers such as PCA. In the study of Pittman, metaplasia, full-thickness chronic inflammation, and/or oxyntic destruction were found as early unspecific changes [33]. Furthermore, hyperplasia of endocrine cells is an early finding in AIG [31]. After progression of disease, a diffuse lymphoplasmatic infiltration of the lamina propria with marked atrophy of the oxyntic glands is found. Intestinal metaplasia becomes noticeable. End stage of disease is defined by distinct reduction or total loss of oxyntic glands; furthermore, pseudopolyps or hyperplastic polyps can be found as well as pancreatic or intestinal metaplasia. On the contrary, the inflammatory reaction is reduced in comparison to earlier stages of disease ([30]; Fig. 2). Immunohistochemical staining of G cells (gastrin) might help to identify the site of biopsy if samples were not sampled separately or labeled correctly, as diagnosis of AIG can only be made from corpus or fundus biopsies.

Measurement of gastric acid might be useful to diagnose early stages of AIG when histological changes are minimal but hypo- or achlorhydria is already present. However, so far there is no technically mature method available [34]. Tube tests are invasive, uncomfortable, and time consuming. Endoscopic sampling of gastric secretion after stimulation with gastrin and further determination of hydrogen ion concentration by titration is accurate but also time consuming and requires technical equipment [35]. However, this method has subsequently been used in several studies [34]. Simple intragastric pH measurement during gastroscopy from aspirates would be quick and cheap, but concerns have been raised that this does not reflect gastric acid secretion or volume of acid secretion [34, 36]. Further studies are needed to test the efficacy and benefit of gastric pH or acid measurement in AIG.

The most sensitive serum biomarker for AIG is PCA [37]. In the past, an immunofluorescence method was used; however, identification of the gastric H/K ATPase as the molecular target for PCA has led to an ELISA, which is more sensitive [38–40]. Intrinsic factor antibodies have proved to be more specific than PCA; however, sensitivity is very low [41] but rises during progression of disease [42]. A combination of PCA and intrinsic factor antibody [41] with anti-Helicobacter pylori and serum gastrin [42] has been suggested. In the study of Antico, levels of PCA did not correlate significantly with severity of disease [42]. However, in other studies [43], PCA showed a trend towards increase over time with a consecutive decrease, which is probably due to destruction of the targeted organ with progression of disease, as also seen in other autoimmune diseases [44]. Furthermore, these specific autoantibodies can precede clinical symptoms by years, as demonstrated for several other autoimmune disorders [43, 45–47]. Gastrin and pepsinogen levels are not specific for diagnosis of AIG but predict the level of atrophy. A combination of autoantibodies and gastrin and pepsinogen levels is the so-called GastroPanel test (Biohit, Helsinki, Finland), a noninvasive (ELISA) test for “serological biopsy” for diagnosis of antral, corpus, or multifocal atrophy. Although theoretically quite promising, data in clinical experience are controversial [48–50]; therefore a recommendation for routine screening use cannot be made.

## Cancer and AIG

As for other chronic inflammatory diseases such as hepatitis, colitis, and pancreatitis, patients with AIG have a higher risk of developing cancer within the chronically inflamed tissue. Correa described already in 1988 the possible pathway of gastric cancer development (Correa hypothesis/pathway): chronic inflammation leads to atrophy of the tissue, which is further followed by intestinal metaplasia. This is considered as a precursor lesion [51]. Unknown genetic, metabolic, or environmental triggers lead to the adenocarcinoma sequence, which is also known from colonic cancer [52]. In a meta-analysis in 2012, an annual incidence of gastric adenocarcinoma of 0.27 % per person-year, with an overall relative risk of 6.8 (95 % CI 2.6–18.1) has been shown [53]. Additionally, chronic achlorhydria increases production of gastrin by G‑cells in the antrum, which then stimulates enterochromaffin cells leading to their hyperplasia. Hyperplasia can further develop into gastric carcinoids [54, 55]. Although this is a rare cause (<1 %) of gastric neoplasia [56], it has been shown that 50 % of patients with gastric carcinoid tumors suffer from pernicious anemia [57].

The American Society for Gastrointestinal Endoscopy recommended in 2006 a single endoscopic evaluation for neoplastic lesions after diagnosis of AIG but no routine follow-up [58]. For patients with only simple, linear, or micronodular hyperplasia, a 3- to 5‑year gastroscopic surveillance interval has been suggested [59]; however, if lesions are more extensive, a closer interval might be needed. If extensive atrophy and/or intestinal metaplasia is present, guidelines suggest triannual surveillance gastroscopy; in patients with adenomas or low-grade dysplasia, annually [30, 51]. However, these guidelines are not specific for patients with AIG but for atrophic gastritis in the setting of Helicobacter pylori infection. Particular guidelines for AIG patients are missing.

In summary, AIG is a common cause of iron deficiency. Despite this, it is not mentioned in guidelines for diagnosis of iron deficiency [60, 61] although it has a great impact on therapy of iron deficiency, as oral iron therapy, which is first-line therapy in many countries, is often ineffective. As there is no therapy for AIG itself, patients need to be monitored carefully lifelong for recurrence of iron deficiency and development of vitamin B12 deficiency. Also, the higher risk of gastric neoplasia should not be forgotten.

## Abbreviations

AIG: autoimmune gastritis
PCA: parietal cell antibodies
